# Reliability and clinical utility of heroin craving questionnaire factors in treatment-enrolled individuals with opioid use disorder

**DOI:** 10.1016/j.drugalcdep.2025.112753

**Published:** 2025-06-07

**Authors:** April C. May, Breanna A. McNaughton-Long, Chrysantha B. Davis, Abigail J. Pleiman, Carmen Buchfink, Rayus Kuplicki, Hung-Wen Yeh, Martin P. Paulus, Jennifer L. Stewart

**Affiliations:** a University of California, San Diego, Department of Psychiatry, United States; b Laureate Institute for Brain Research, United States; c Health Services & Outcomes Research, Children’s Mercy Research Institute, Kansas City, MO, United States; d University of Missouri Kansas City School of Medicine, Kansas City, MO, United States; e Oxley College of Health and Natural Sciences, University of Tulsa, United States

**Keywords:** Craving, Opioid use disorder, Heroin craving questionnaire, Psychometrics, Clinical utility

## Abstract

Psychometric properties of opioid craving assessments are underexamined, limiting their potential utility for treatment studies of opioid use disorder (OUD). To address this gap, the current study aimed to evaluate the predictive utility of the Heroin Craving Questionnaire (HCQ) for future substance use outcomes. Treatment-enrolled participants with OUD (*n* = 128) completed the Heroin Craving Questionnaire (HCQ) at baseline and were followed up to assess future return to use versus abstinence. Individuals who maintained abstinence at each of three follow-up visits also completed the HCQ again. Exploratory factor analysis (EFA) was performed on baseline HCQ items, and extracted factors were evaluated for: (1) internal consistency reliability (Cronbach’s alpha); (2) associations with demographic and clinical variables; (3) follow-up group differences; (4) test-retest reliability (intraclass coefficients: ICCs); and (5) change across visits. EFA results produced a three-factor structure, retaining 30 of 45 HCQ items and accounting for 54.60 % of the variance: (1) Lack of Self-Control (10 items, alpha=.93); (2) Positive Expectancies (11 items, alpha=.91); and (3) Urgency (9 items, alpha=.89). Results indicated that: (1) elevated scores on all three factors were associated with comorbid amphetamine use disorder; (2) higher Lack of Self-Control and Positive Expectancies scores related to fewer days since last heroin use; (3) greater Lack of Self-Control and Urgency scores were associated with higher anxiety severity; and (4) ICCs showed moderate to good test-retest reliability between baseline and follow-up visits. However, factor scores at baseline could not differentiate future relapsers versus abstainers. More research is warranted to replicate these factors in additional OUD samples.

## 1. Introduction

Traditionally defined as the subjective experience of wanting to use a specific substance, craving is a complex phenomenon influenced by physiological factors, environmental cues, and psychological conditions (Bergeria et al., 2023; Tiffany and Wray, 2012). Although the construct of craving is highly debated, it is conceptualized to play a crucial role in the development and maintenance of substance use disorders (SUD), and addressing drug craving is believed to be vital for effective treatment and long-term recovery (Bergeria and Strain, 2022; Shmulewitz et al., 2023; Vafaie and Kober, 2022). An accurate understanding of the role of craving in SUD is crucial to improving our ability to identify risk factors for harmful drug use and to develop effective prevention and intervention programs (McHugh et al., 2021). One limiting factor to our understanding of the full clinical utility of craving is our ability to consistently and precisely measure this construct.

Several substance-specific measures have been developed to evaluate craving, resulting in a lack of standardized measurement and limited ability for between-study comparisons (Goodyear and Haass-Koffler, 2020). For instance, a recent review identified 15 opioid-specific measures used across 87 studies (Kleykamp et al., 2019). Unidimensional measurement of craving via a single-item visual analog scale (VAS) was most commonly used (in 41 out of 87 studies). In contrast, multidimensional assessments of craving, such as the Desires for Drug Questionnaire and the Heroin Craving Questionnaire (HCQ), were only used in 12 and 10 of 87 studies, respectively. While the possibility of measuring craving with a single VAS scale is appealing given the low patient burden, high face validity, and ease of use for repeated assessments, it has been argued that this metric does not sufficiently capture the complex construct of craving (Heinz et al., 2006; Tiffany and Wray, 2012). The HCQ, a standardized self-report measure of the intensity and frequency of heroin-related craving (Tiffany et al., 1993a), was specifically developed as a multidimensional alternative to single-item craving measures.

Evolving from the Cocaine Craving Questionnaire (Tiffany et al., 1993b), the 45 items of the HCQ were developed to represent five theoretical conceptualizations of drug craving: desire to use, intent to use, anticipation of positive outcome, anticipation of relief from withdrawal, and lack of control over use (Tiffany et al., 1993a). Participants respond to each item using a Likert-type scale ranging from 1 (strongly disagree) to 7 (strongly agree). Item responses from 230 non-treatment seeking individuals actively using heroin were entered into an exploratory factor analysis, resulting in four first-order factors represented by 34 of the 45 items (Desire = 14 items, Lack of Self-Efficacy = 9 items, Relief = 7 items, Compulsivity = 4 items; see Heinz et al., 2006), as well as one higher order factor possessing excellent internal consistency reliability (Cronbach’s alpha =.94). Although only 34 items contributed to the underlying HCQ factor structure, all 45 items were retained in the final measure (Tiffany et al., 1993a).

To date, only one additional psychometric evaluation of the HCQ is known. This study validated a Greek-language version in a sample of 258 patients receiving methadone or buprenorphine for opioid maintenance treatment, demonstrating strong internal consistency, as well as construct and predictive validity (Leventelis et al., 2020). However, the broader psychometric literature on the HCQ remains limited. It is still unclear whether items of each original HCQ subscale measure the same latent craving constructs or demonstrate stability over time in samples with varying characteristics (e.g., individuals actively using heroin versus treatment-enrolled individuals with or without maintenance on medications for opioid use disorder [OUD/MOUD]).

Studies examining clinical associations with the HCQ suggest that current heroin use, lower MOUD doses, and drugs precipitating opioid withdrawal are associated with higher HCQ scores. For individuals reporting heroin as their drug of choice (*n* = 101), higher 34-item total and Desire HCQ scores at the outset of treatment were associated with fewer opioid-negative urine tests during treatment (Heinz et al., 2006). With respect to MOUD dosing, among 15 individuals on buprenorphine maintenance for OUD, participants who were actively using heroin endorsed higher scores on the 34-item HCQ total score than those who had recently abstained (Greenwald et al., 1999). In contrast, naloxone injection (precipitating opioid withdrawal) resulted in greater HCQ 34-item total and Desire scores than saline injection among nine methadone-maintained individuals with OUD (whereas a VAS craving score indicated no difference between conditions; Schuster et al., 1995). Although the 34-item HCQ total score and the HCQ Desire subscale appear to be linked to opioid withdrawal and opioid-positive drug tests, additional research is warranted to determine whether HCQ factors can predict abstinence versus return to use or track changes with abstinence during early recovery from OUD.

### 1.1. The present study

This study aimed to examine the factor structure of HCQ items in a treatment-enrolled sample of individuals with OUD and subsequently examine the extracted factors for internal consistency reliability and associations with demographic and clinical variables. In addition, test-retest reliability and changes throughout early OUD recovery were investigated across four HCQ assessment visits in individuals who successfully remained abstinent. Analogous analyses were conducted on the four original HCQ factors reported in the literature (Heinz et al., 2006; Tiffany et al., 1993a) for comparison.

## 2. Methods

### 2.1. Participants

Data for this secondary analysis were collected as part of a naturalistic longitudinal study (“Plasticity of Aversive Salience in Opioid Use Disorder” [PASO]). PASO study primary aims were to identify self-report, behavior, and brain metrics of treatment-enrolled individuals with OUD that: (1) predicted return to use during early recovery (the first three months after study enrollment); and (2) changed over the course of early recovery when compared to healthy individuals. Healthy adults also enrolled in the PASO study (*n* = 46) had never used heroin and are not included here. A total of 183 treatment-enrolled individuals reporting opioids as their current drug of choice were recruited from one of two addiction treatment centers in Tulsa, Oklahoma: (1) GRAND Addiction Recovery Center, which provides 30–60 day residential treatment, followed by transitional sober living opportunities; and (2) Women In Recovery (WIR), a criminal diversion program for females providing 17-month residential and intensive outpatient treatment.

Study inclusion criteria were: (1) a DSM-5 diagnosis of past-year OUD; (2) age between 18 and 55 years; and (3) English proficiency. Study exclusion criteria were: (1) DSM-5 psychotic disorders (including schizophrenia and bipolar I disorder); (2) non-corrected vision or hearing issues; (3) significant and unstable medical issues; and (4) moderate to severe traumatic brain injury. To assess OUD group inclusion and exclusion criteria, individuals consented to a screening visit, wherein they: (1) provided demographic, treatment onset, drug use recency, drug use history, and current medication information; (2) completed the Mini International Neuropsychiatric Interview (MINI) for DSM-5 (Sheehan et al., 2016); and (3) provided a medical history. For the present analysis, we included only participants who endorsed 50 or more lifetime heroin use sessions at screening (*n* = 128). Screening and study visits took place at the Laureate Institute of Brain Research (Tulsa, OK) and both screening and study human subjects protocols were approved by Western IRB. All data were collected from May 2021 to October 2024.

### 2.2. Procedure

Eligible individuals provided informed consent to participate in the study, which included a baseline visit (visit 1) as well as three additional follow-up visits (visits 2, 3, and 4) spaced approximately one month apart, if participants met specific criteria. Participants were informed that follow-up visit eligibility was contingent on ongoing abstinence from all alcohol, cannabis, and illicit drug use with the exception of nicotine. This status was confirmed via self-report, breathalyzer, and urine drug screen at the beginning of each visit for participants who arrived at follow-up visits. Consent was gathered for researchers to engage with designated secondary contacts to gain information about sobriety status and possible return to use. Abstinence versus return to use was documented for each participant across the follow-up window.

Return to use was defined as any use of illicit substances or alcohol following study enrollment. This broader definition was selected to reflect the high prevalence of polysubstance use among individuals with OUD, as supported by prior studies demonstrating that most individuals with OUD also engage in concurrent use of other substances (Bourgault et al., 2022; Crummy et al., 2020). Given that return to use in real-world settings often involves resumption of multiple substances—not solely opioids—this approach was intended to capture clinically meaningful lapses in abstinence and recovery. This definition also allowed for more robust detection of return to use events, particularly given the multiple sources of follow-up data and sample size constraints. While this approach may reduce specificity in linking resumption of use directly to opioid use or craving, it offers a pragmatic and ecologically valid measure of return to use.

**Fig. 1.**
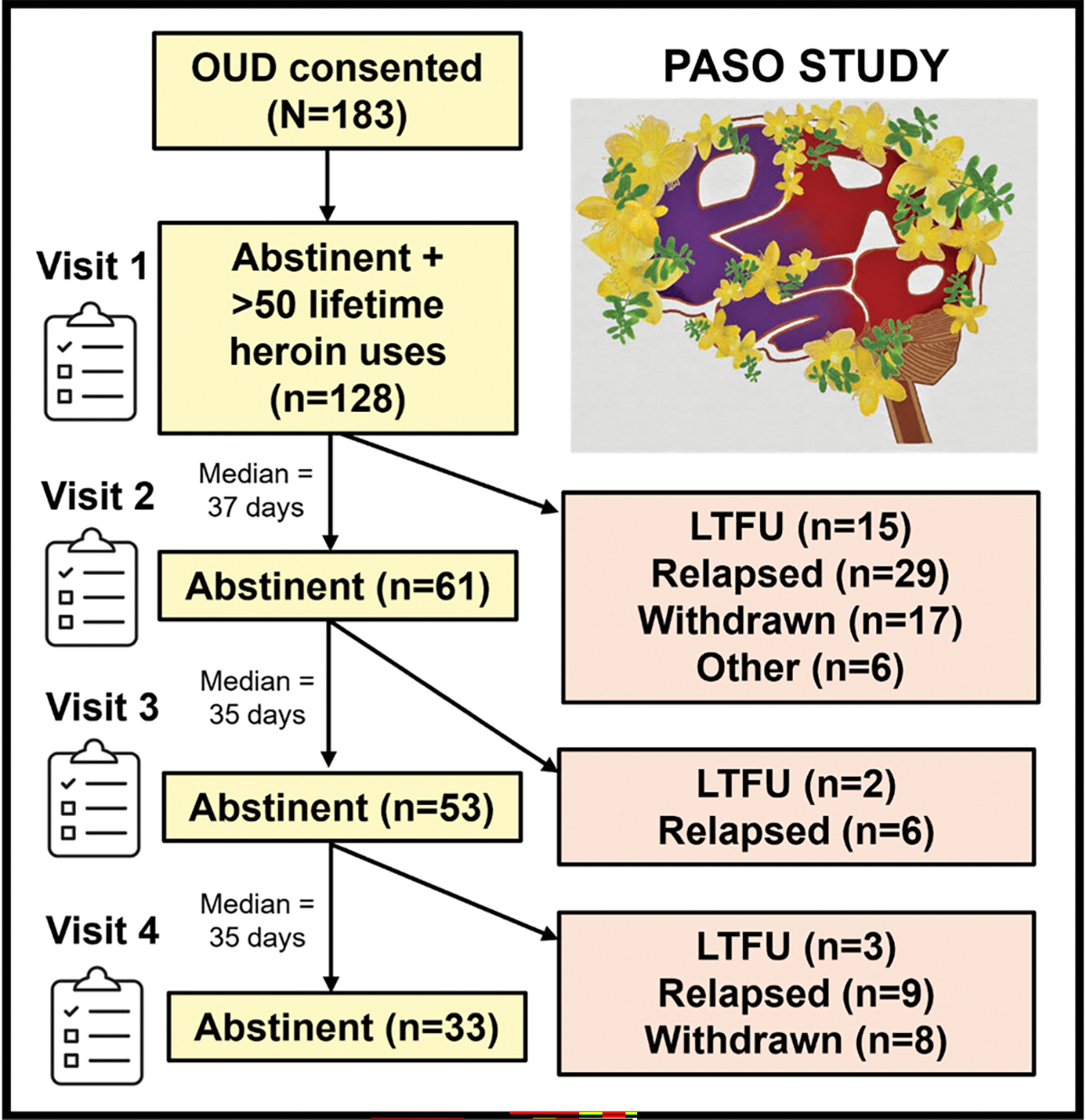
Overview of plasticity of aversive salience in opioid use disorder (PASO) study data collection at each of four visits. Participants with opioid use disorder (OUD) completed the heroin craving questionnaire at up to four visits spaced approximately one month apart as long as abstinence was documented at the start of each visit (via self-report and urine drug screen/breathalyzer). LTFU = lost to follow-up. other = treatment site disciplinary action unrelated to substance use resulting in study termination. Duration between completed visits is illustrated by the median (Visit 1 to Visit 2 range: 24–105 days; Visit 2 to Visit 3 range: 30–126 days; Visit 3 to Visit 4 range: 25–56 days).

Each visit consisted of questionnaire, behavioral, neuroimaging, and blood draw sessions unless contraindications were reported (e.g., metal in body for a magnetic resonance imaging scan). Behavioral, neuroimaging, and blood biomarker data are not reported here. At baseline, participants completed: (1) the Customary Drinking and Drug Use Record (CDDR; Brown et al., 1998) to obtain age of first and regular opioid use, lifetime opioid uses, and drug injection behavior; and (2) the Anxiety Sensitivity Index 3 (ASI-3; Taylor et al., 2007) to measure fear of arousal-related sensations shown to be positively correlated with OUD symptom severity (Stathopoulou et al., 2021). Participants completed the 45-item HCQ (Tiffany et al., 1993a) at visit 1 and each follow-up visit as long as they met abstinence criteria outlined above. They were asked to rate from 1 (strongly disagree) to 7 (strongly agree) how they felt about the following 45 statements reflected by HCQ items (e.g., “I would feel so good and happy using heroin”, “Even if using heroin was possible, I probably wouldn’t use it”). Fig. 1 illustrates participant status at each visit: (1) abstinent; (2) return to use; (3) lost to follow-up (did not respond to emails, calls or texts and/or did not arrive at scheduled visits); (4) withdrawn; or (5) other (treatment site disciplinary action unrelated to substance use resulting in study termination). Complete HCQ data were available for all 128 participants at visit 1. A total of 33 individuals (17 F, 16 M) remained abstinent throughout the study (via self-report and urine/breathalyzer data) and had complete HCQ data across all four visits.

### 2.3. Statistical analysis

Analyses were performed in R Studio (Posit Team, 2024). R version 4.2.1 (packages: *tidyverse*, *ggplot2*, *gmodels*, *car*, *psych*, *stats*, *olsrr*, *GPArotation*, *ez*, and *lm*). EFA analyses were guided by Garson (2023) and additional statistics were guided by Field and colleagues (2012).

#### 2.3.1. Sample demographics and clinical characteristics

Descriptive statistics were calculated for recruitment site, sex, race, ethnicity, education, DSM-5 diagnoses, medications, age, days in treatment, drug use recency, lifetime opioid uses, drug injection behavior, and age of first/regular opioid use across participants.

#### 2.3.2. Exploratory factor analysis (EFA) on HCQ data

Given that the EFA for the original HCQ measure is unpublished (Tiffany et al., 1993a) and an additional EFA by Heinz and colleagues (2006) reported discrepancies between their item factor loadings and those of Tiffany et al. (1993a), we determined that computing an EFA on baseline HCQ data was more appropriate than a confirmatory factor analysis, as the overall craving model remained unclear. Although 11 of the 45 HCQ items (1, 5, 8, 9, 11, 15, 19, 25, 27, 36, and 43) did not significantly load on any of the four original HCQ factors (Heinz et al., 2006; Tiffany et al., 1993a), our EFA included all 45 items. Prior to EFA entry, 20 of the 45 items (2, 4, 5, 6, 7, 9, 12, 21, 25, 26, 27, 28, 31, 32, 33, 35, 36, 38, 44, and 45) were reverse scored by subtracting each score from a value of 8.

Several steps were taken in preparation for EFA. First, the Kaiser-Meyer-Olkin (KMO) measure of sampling adequacy was computed for each of the 45 items to evaluate whether the data were well-suited to factor analysis. Second, histograms, Shapiro-Wilk tests, and multivariate normality tests of skewness and kurtosis were computed for HCQ items. Third, Bartlett’s test of sphericity was computed to determine whether items were significantly correlated. Fourth, the number of eigenvalues greater than 1 illustrated in the scree plot was used to determine the number of factors to be extracted from the data during EFA (using the principal axis factoring solution and oblimin rotation). No items were negatively associated with any factors and items that did not load significantly (and positively) on any factor (> 0.4) were removed and the EFA was then rerun to obtain updated factor loadings. KMO metrics for retained items were then recalculated. EFA analysis was then rerun with the 19 participants from the WIR site removed to evaluate their influence on item loadings and factor structure.

#### 2.3.3. Internal consistency reliability on extracted factors

Cronbach’s alpha was calculated for each factor emerging from the visit 1 EFA data and values ≥ .70 were interpreted as acceptable (Tavakol and Dennick, 2011).

#### 2.3.4. Cross-sectional associations with extracted factors

Multiple linear regressions were computed on visit 1 HCQ data, one for each factor extracted from the EFA. There were eight predictors in each model: (1) age (years); (2) sex (1 = female, 0 = male); (3) education: completed high school or equivalent (1 = yes, 0 = no); (4) past-year diagnosis of amphetamine use disorder, which was the most common comorbid substance use disorder (AMP; 1 = yes, 0 = no); (5) MOUD (1 = yes, 0 = no); (6) lifetime diagnosis of major depressive disorder (MDD), which was the most common comorbid non-substance use disorder; (7) days since last heroin use; and (8) the ASI-3 total score, reflecting anxiety sensitivity symptoms. Recruitment site was not included as a predictor because it was confounded with sex and MOUD. Normality and univariate outliers of predictors and dependent variables were examined using histograms and boxplots. After initial regression models were computed, multivariate outliers were defined by: (1) Cook’s distance values greater than three times the mean value; and (2) standardized residuals identified by the R *plot* function as outliers when graphed against fitted model and/or leverage values. Outliers were removed list-wise from each analysis and regressions were rerun. Durbin-Watson values were all *p* > .05 after outlier removal, confirming uncorrelated model residuals. Histograms also confirmed the residual normality assumption. All variance inflation factor (VIF) values were < 2, indicating that predictors were not multicollinear. To evaluate univariate effects of MOUD group, sex, and recruitment site, Welch’s *t* tests were computed on HCQ factor scores.

#### 2.3.5. Baseline HCQ factors and relapse status at follow-up

Extracted HCQ factors at baseline were compared between participants who resumed use via self-report, breathalyzer, and/or urine drug screen (or whose secondary contact/treatment center reported that they had resumed use) and participants who remained abstinent during the follow-up period. Histograms and Levene’s test evaluated univariate normality and homogeneity of variance; if one or both of these assumptions were violated for these factors, data were square root-transformed. Welch’s *t* tests then compared group differences for each factor.

#### 2.3.6. Test-retest reliability on extracted factors

Intraclass coefficient (ICC) estimates were computed for new HCQ factor scores for all participants who remained abstinent at each visit (illustrated in Fig. 1): between visits 1 and 2 (*n* = 61), visits 1 and 3 (*n* = 53), and visits 1 and 4 (*n* = 33). Two-way mixed effects models were computed (one for each of the three extracted factor scores comparing visit 1 to each follow-up visit), with subject as a random factor and a single measurement across visits as a fixed factor (as repeated measurements cannot be considered randomized samples), with analysis based on the absolute agreement between measurements (Koo and Li, 2016; this model corresponds to the ICC3k result for the *ICC* function within the R *psych* package).

#### 2.3.7. Changes in extracted factor scores as a function of visit

For participants who remained abstinent across all four visits (*n* = 33), three repeated-measures analysis of variance (ANOVA) models were computed (one for each of the three extracted factor scores) with visit (V1, V2, V3, V4) as the repeated measure and score as the dependent variable. For violations of the sphericity assumption indicated by Mauchly’s test, a Greenhouse Geisser (GG) or Huynh-Feldt (HF) correction was applied where appropriate. Significant main effects of visit were followed up by post-hoc pairwise Bonferroni-corrected *t*-tests. Partial eta-squared (*η*^*2*^) is reported as a measure of effect size.

#### 2.3.8. Analysis of the four original HCQ factors

Individual items within each of the four original HCQ factors (Tiffany et al., 1993a, outlined by Heinz et al., 2006) were evaluated for internal consistency reliability at visit 1 and then summed to compare test-retest reliability by calculating ICCs across visits: (1) Desire (14 items: 10, 14, 18, 20, 22, 23, 24, 30, 34, 37, 39, 40, 41, and 42); (2) Lack of Self-Efficacy (9 items: 2, 4, 6, 21, 26, 28, 31, 35, and 38); (3) Relief (7 items: 3, 7, 12, 13, 17, 32, and 33); and (4) Compulsivity (4 items: 16, 29, 44, and 45). Linear regressions identified factor associations with demographic and clinical variables and repeated-measures ANOVAs investigated potential changes in factor scores across visits.

## 3. Results

### 3.1. Sample demographics and clinical characteristics

Table 1 indicates that within the sample, 36 % were female and 77 % had completed high school (or equivalent). On average, participants were over 30 years of age, started using opioids regularly at age 19, reported being abstinent under one month prior to treatment onset, and completed the baseline visit over one month after treatment onset (so mean abstinence from alcohol and any non-prescribed drug other than nicotine and caffeine was approximately two months in duration). A total of 80 % reported injecting opioids, 85 % endorsed using opioids more than 1000 sessions in their lifetime, and over 90 % reported heroin and/or fentanyl as their current opioids of choice. In addition to demographic information, Table 1 highlights clinical comorbidity and prescribed medication use. The most common comorbid diagnoses were past-year amphetamine use disorder (AMP; 63 %), past-year cannabis use disorder (24 %), and lifetime MDD (24 %), while the most frequently prescribed medications were buprenorphine (71 %) and antidepressants (48 %).

Of the 128 participants, Fig. 1 illustrates that 33 (26 %) remained abstinent and had completed all four visits, 44 (34 %) had returned to use, 25 (20 %) withdrew (the most common reasons were moving >30 miles away and/or working a full-time job), 20 (16 %) were lost to follow-up, and 6 (4 %) were excluded from further visits (i.e. left treatment early, went to jail). Return to use was determined via: (1) secondary contact/treatment center information (*n* = 11, substances unknown); (2) self-report (*n* = 18; 8 alcohol, 6 opioids, 5 cannabis, 4 amphetamine, 2 unknown, 1 sedatives); (3) urinary drug screen (*n* = 14; 5 amphetamine, 4 cannabis, 3 opioids, 1 sedatives, and 4 unknown [their urine was falsified]); and (4) breathalyzer (*n* = 1).

### 3.2. EFA on visit 1 HCQ data

Histograms of individual items (Figure S1) indicated that most distributions were positively skewed (indicating lower craving). Six items with KMO values less than.70 were removed prior to EFA (item 8 =.60, item 9 =.31, item 29 =.61, item 32 =.68, item 36 =.67, item 44 =.48). For the 39 remaining items with KMO values > .70, the overall KMO value was.91, indicating adequate sampling for EFA. Univariate and multivariate normality were violated (all Shapiro-Wilk tests *p* < .05; multivariate skew = 14852.18 and kurtosis = 17.42, both *p* < .05). However, normality is not a critical assumption of EFA if no significance testing is interpreted (Garson, 2023). Nonparametric polychoric correlations were unable to be substituted for Pearson’s correlations within the EFA because the same number of score values (1 through 7) were not present across items.

Eigenvalues from the scree plot suggested a three-factor solution to the data (factor 1 = 15.17, factor 2 = 2.64, factor 3 = 1.47). Bartlett’s test of sphericity indicated that HCQ items were significantly correlated (*p* < .05); therefore, EFA was computed using the principal factor solution with three factors, oblimin rotation to address item intercorrelations, and a factor loading score cutoff of 0.4. Eight items (3, 11, 16, 21, 25, 35, 39, and 42) did not meet the cutoff for any factor. The EFA was repeated, removing these items, which resulted in item 5 no longer meeting the cutoff; once the EFA was rerun removing item 5, the remaining items all met the cutoff for one factor. No items were cross-loaded to multiple factors. A total of 30 HCQ items were retained (final KMO =.91; range of individual KMO items =.78 –.95) and the three factors accounted for 54.60 % of the total variance. Moreover, the degrees of freedom-corrected root mean square of the residuals was 0.05, indicating a good fit. Table S1 lists the 15 original HCQ items that were not retained.

**Fig. 2.**
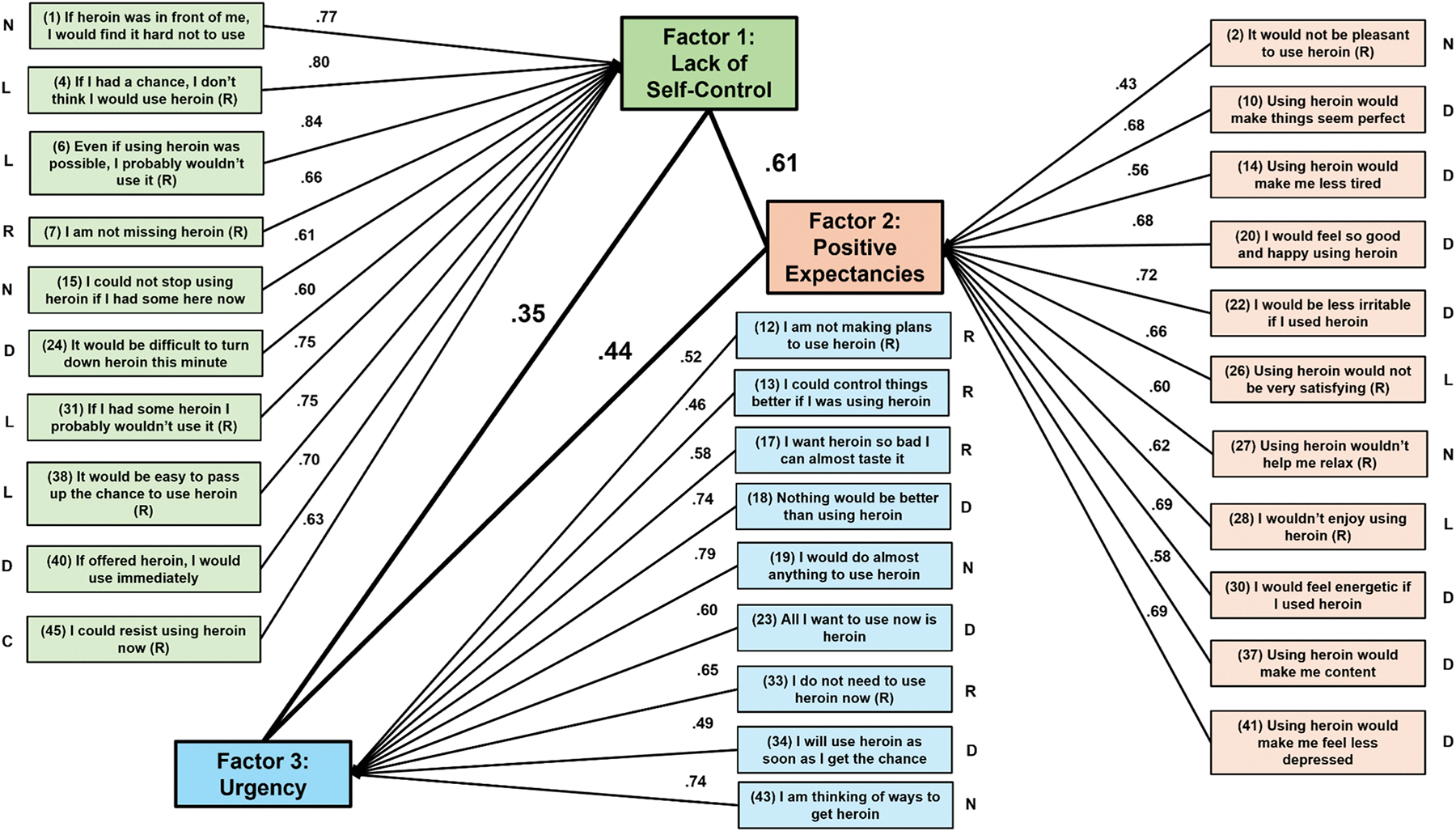
Exploratory factor analysis item loadings from baseline heroin craving questionnaire (HCQ) data (*n* = 128). (R) = reverse scored prior to factor analysis. Lines between items and factors indicate factor loadings, whereas darker lines between factors indicate correlations. The letter *n*ext to each item reflects its original HCQ factor loading: d = desire, l = lack of Self-Efficacy, r = relief, c = compulsivity, or *n* = *n*one.

Fig. 2 shows the individual item loadings for each of the three new factors as well as their original HCQ factor loading. Notably, items within these new factors did not fully align with any of the four original HCQ factor scores. A review of item content suggested that: (1) Factor 1 (10 items) reflected lack of self-control over heroin use (and included 4 items from the original HCQ Lack of Self-Efficacy subscale); (2) Factor 2 (11 items) reflected positive expectancies of heroin use (and included 7 items from the original HCQ Desire subscale); and (3) Factor 3 (9 items) reflected urgency to use heroin (and included 3 and 4 items each from the original HCQ Desire and Relief subscales, respectively). Lack of Self-Control correlated.61 with Positive Expectancies and.35 with Urgency, whereas Positive Expectancies and Urgency correlated at.44. The EFA run on participants from the GRAND site alone (*n* = 109) replicated the pattern of item loadings and factor structure of the EFA on the entire sample, with the exception that item 37 loaded on two factors: Positive Expectancies (.50) and Urgency (.40) (see Figure S2).

### 3.3. Analysis of extracted HCQ factors

#### 3.3.1. Internal consistency reliability

Cronbach’s alpha was excellent for Lack of Self-Control (.93) and Positive Expectancies (.91) and good for Urgency (.89).

#### 3.3.2. Cross-sectional associations

**Fig. 3.**
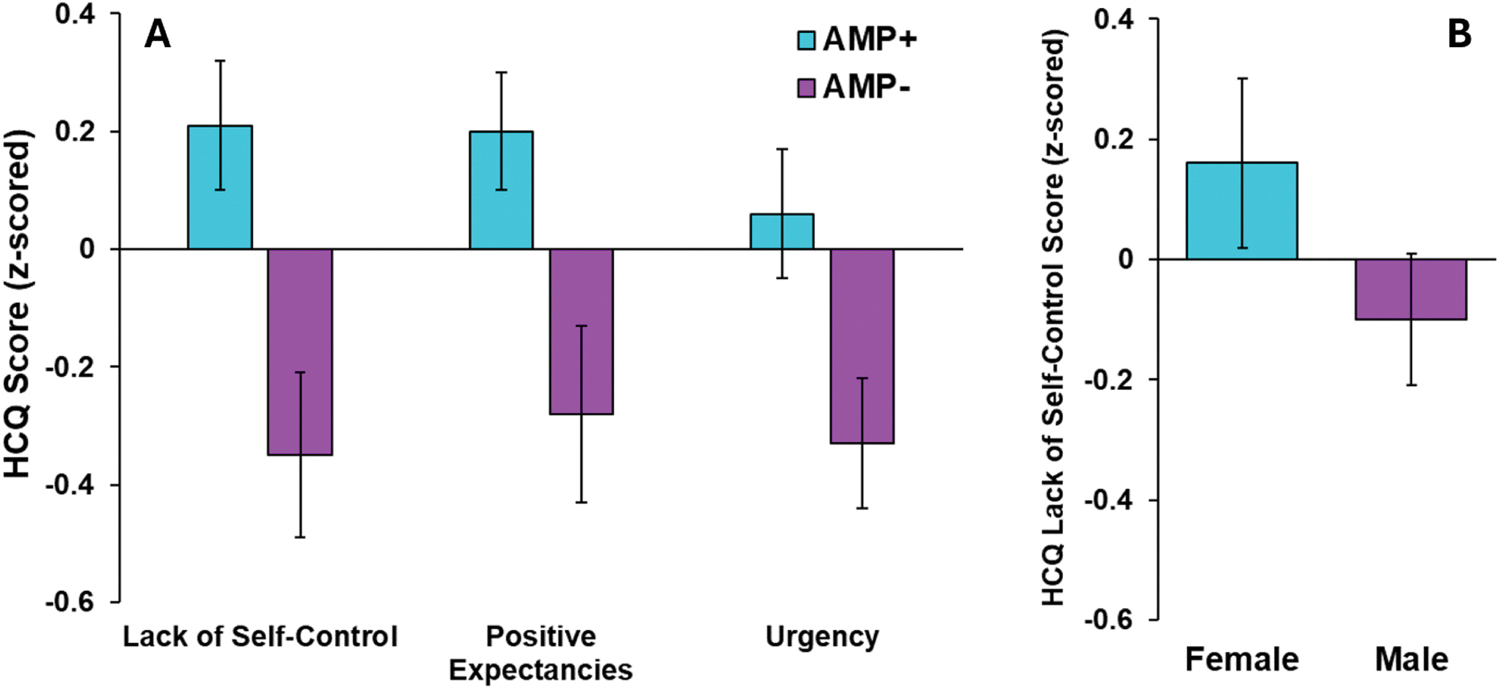
Significant regression predictors of HCQ factors: (A) participants with comorbid amphetamine use disorder (AMP+) exhibited higher HCQ lack of self control, positive expectancies, and urgency scores than those without AMP (AMP-); and (B) females reported higher HCQ lack of Self-Control scores than males. Graphs reflect non-residualized group means and standard errors.

**Fig. 4.**
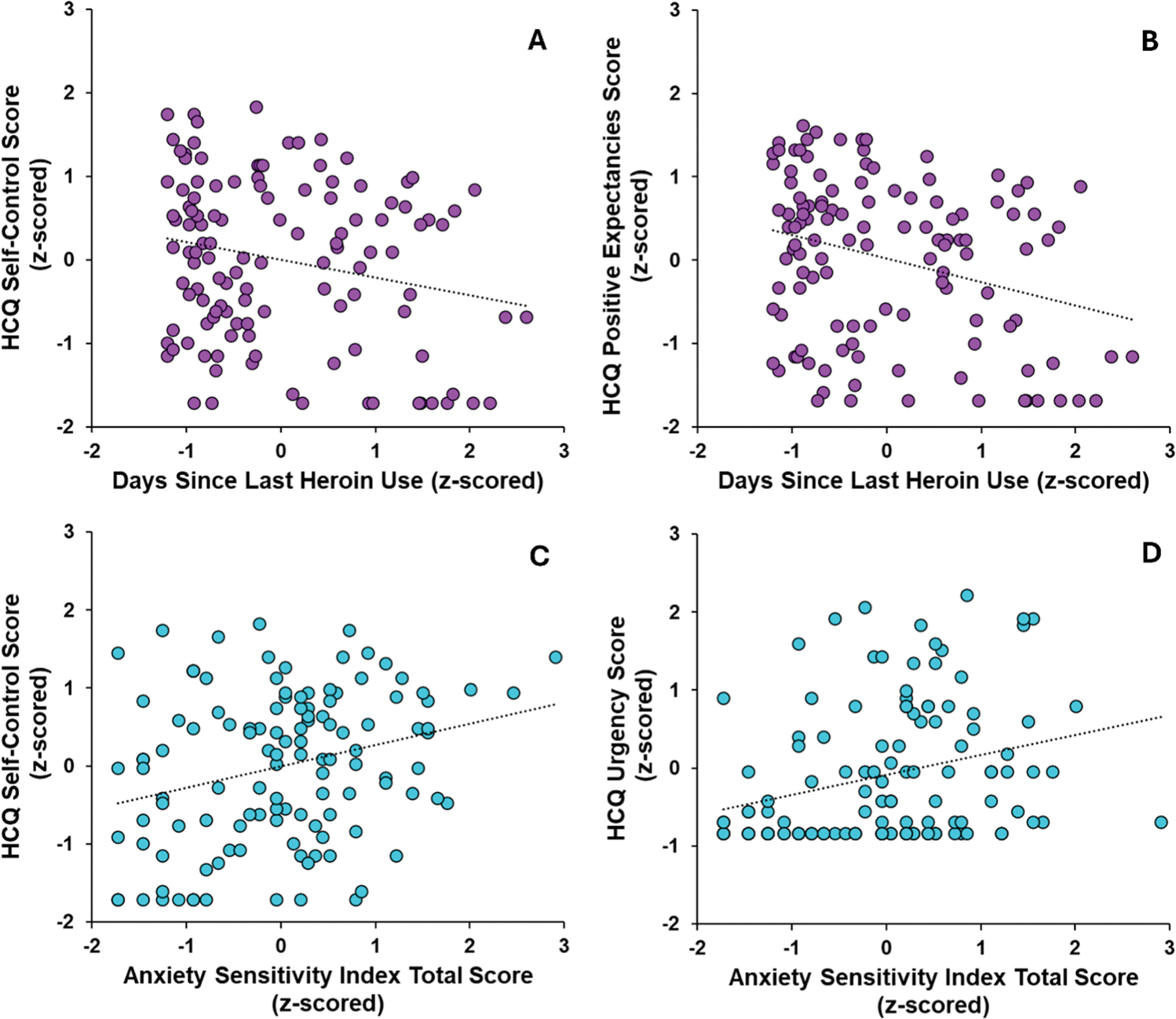
Significant regression predictors of HCQ factors: fewer days since last heroin use was associated with (A) higher HCQ lack of Self-Control scores and (B) higher HCQ positive expectancies scores. Greater anxiety sensitivity index total scores were associated with (C) higher HCQ lack of Self-Control scores and (D) higher HCQ urgency scores. Graphs reflect non-residualized scores.

The three dependent variables (Lack of Self-Control, Positive Expectancies, and Urgency) were all square root transformed, while days since last heroin use was log transformed and ASI-3 total score was square root transformed. All predictors were z-scored prior to regression entry. Table 2 presents statistics for overall regression models as well as individual predictors within each model. Models were significant for all three extracted factors (*p* ≤.001). Fig. 3A illustrates that participants with comorbid AMP reported higher Lack of Self-Control, Positive Expectancies, and Urgency scores than participants without AMP, whereas Fig. 3B demonstrates that females endorsed higher Lack of Self-Control scores than males. Moreover, fewer days since last heroin use was associated with higher scores for Lack of Self-Control (Fig. 4A) and Positive Expectancies (Fig. 4B). Lastly, greater ASI-3 anxiety scores related to higher scores for Lack of Self-Control (Fig. 4C) and Urgency (Fig. 4D). Univariate analyses indicated that HCQ factor scores did not differ as a function of MOUD status, sex, or site (Table S2).

#### 3.3.3. Baseline HCQ factors and relapse status at follow-up

Figure S3 indicates that participants who returned to use during the follow-up window (*n* = 44) did not differ from participants who remained abstinent (*n* = 33) on baseline scores for Lack of Self-Control (*t*[74] = −0.001, *p* = .99), Positive Expectancies (*t*[75] = 1.34, *p* = .18), or Urgency (*t*[73]=−0.05, *p* = .96). Removal of participants whose return to use status was based solely on secondary contact information (*n* = 11) did not impact results (Lack of Self-Control *p* = .77, Positive Expectancies *p* = .08, and Urgency *p* = .41).

#### 3.3.4. Test-retest reliability

ICC estimates listed in Table 4 indicated that Lack of Self-Control, Positive Expectancies, and Urgency showed moderate to good reliability across visits (ICC range =.54 to.82).

#### 3.3.5. Changes in scores over time

**Fig. 5.**
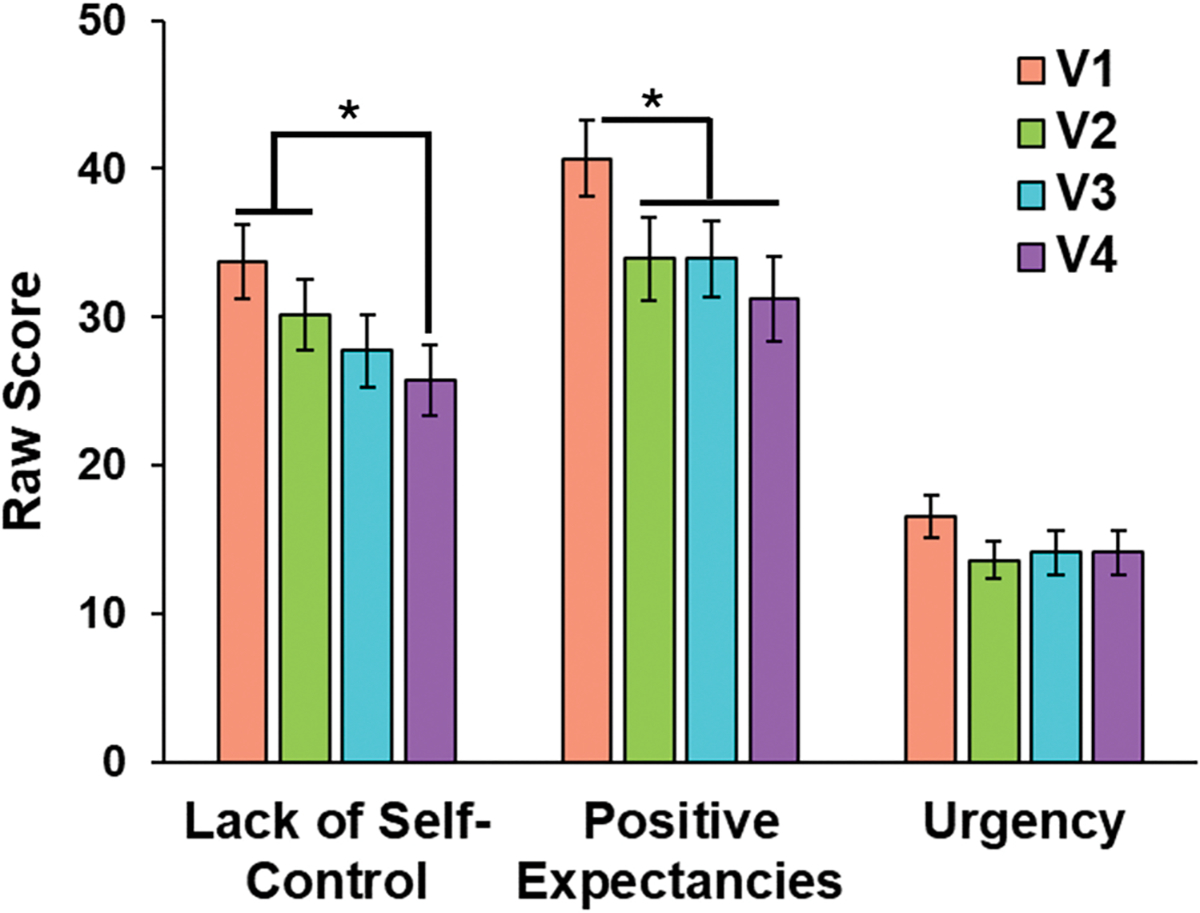
Extracted heroin craving questionnaire (HCQ) factor scores as a function of visit (V1, V2, V3, V4) in participants who remained abstinent across visits (*n* = 33). participants reported lower lack of Self-Control scores at visit 4 than visits 1 and 2 (p = .01 and.03) and lower positive expectancies scores at visits 2, 3, and 4 than visit 1 (p = .03,.047, and.003). error bars reflect + 1 standard error. Asterisks indicate significant differences between visits.

Fig. 5 illustrates that significant main effects of visit emerged for Lack of Self-Control (*F*(3, 96) = 6.70, GG-corrected *p* = .003, *η*^*2*^ = .04) and Positive Expectancies, (*F*(3, 96) = 7.79, uncorrected *p* < .001, *η*^*2*^ = .05), such that participants who maintained abstinence across study visits (*n* = 33) reported lower Lack of Self-Control scores at visit 4 than visits 1 and 2 (*p* = .01 and.03) and lower Positive Expectancies scores at visits 2, 3, and 4 than visit 1 (*p* = .03,.047, and.003). In contrast, no visit effect emerged for Urgency (GG-corrected *p* = .11).

### 3.4. Analysis of the four original HCQ factors

#### 3.4.1. Internal consistency reliability

Cronbach’s alpha was excellent for Desire (.93) and good for Lack of Self-Efficacy (.87) but unacceptable for Relief (.67) and Compulsivity (.62).

#### 3.4.2. Cross-sectional associations

Overall regression models were significant for all factors (*p* ≤.01; Table 3). Desire and Relief scales were square root transformed prior to regression entry due to non-normality. Table 3 demonstrates that (1) higher Desire, Lack of Self-Efficacy, and Relief scores were associated with comorbid AMP; (2) greater Desire scores related to higher ASI-3 scores; (3) higher Lack of Self-Efficacy and Relief scores were associated with comorbid MDD; and (4) greater Desire, Lack of Self-Efficacy, Relief and Compulsivity scores related to fewer days since last heroin use. Univariate analyses indicated that participants on MOUD reported lower Compulsivity scores, but HCQ factor scores did not differ by sex or site (Table S2).

#### 3.4.3. Baseline HCQ factors and relapse status at follow-up

Follow-up group analyses depicted in Figure S4 indicated that participants who returned to use and abstinent participants did not differ on scores for Desire (*t*[75] = 0.77, *p* = .44), Lack of Self-Efficacy (*t*[74] = 0.08, *p* = .94), Relief (*t*[75] = 0.11, *p* = .91), and Compulsivity (*t*[72] = −0.70, *p* = .48). Removal of participants whose return to use status was based solely on secondary contact information (*n* = 11) did not impact results (Desire *p* = .18, Lack of Self-Efficacy *p* = .59, Relief *p* = .47, and Compulsivity *p* = .75).

#### 3.4.4. Test-retest reliability

ICC estimates listed in Table 4 indicated that Desire, Lack of Self-Efficacy, Relief, and Compulsivity showed moderate to good reliability across visits (ICC range =.55 to.78).

#### 3.4.5. Changes in scores over time

Figure S5 illustrates that a significant main effect of visit emerged for Desire (*F*(3, 96) = 8.45, uncorrected *p* < .001, *η*^*2*^ = .07) but not Lack of Self-Efficacy (*p* = .07), Relief (*p* = .12) or Compulsivity (*p* = .34). Participants reported lower Desire scores at visits 2, 3, and 4 compared to visit 1 (*p* = .02, *p* = .007, and *p* = .004, respectively).

## 4. Discussion

To address crucial gaps in the literature, the present study evaluated the factor structure, reliability and clinical utility of the HCQ in treatment-enrolled individuals with OUD who were abstinent for approximately two months at the time of their baseline visit. Our EFA produced three factors (Lack of Self-Control, Positive Expectancies, and Urgency) that demonstrated good to excellent levels of internal consistency reliability and exhibited moderate to good levels of test-retest reliability during early recovery in individuals who maintained abstinence. Two out of the three factors (Lack of Self-Control and Positive Expectancies) also showed decreases as a function of abstinence duration, suggesting lower anticipated positive outcomes of heroin use and greater perceived self-control over heroin use during early recovery; these craving reductions with abstinence align with a recent review of studies in clinical populations (Bergeria et al., 2024).

With respect to clinical associations, higher Lack of Self-Control and Positive Expectancies scores were linked to fewer days since last heroin use, replicating prior work showing that craving is stronger for individuals with more recent drug consumption (e.g., Zilberman et al., 2007). In addition, individuals with comorbid AMP reported higher Lack of Self-Control, Positive Expectancies, and Urgency scores than those without AMP, replicating research indicating that the presence of opioid and amphetamine polysubstance use amplifies drug craving (Hochheimer et al., 2023). Moreover, higher Lack of Self-Control and Urgency scores were associated with greater anxiety sensitivity symptom severity, findings consistent with prior work showing that anxiety sensitivity moderates state craving levels within an OUD sample (Stathopoulou et al., 2018).

However, extracted HCQ factors at baseline did not significantly differentiate individuals who resumed use from those who remained abstinent during the follow-up period, suggesting limited predictive clinical utility. On the whole, fewer items (30) than the entire 45-item HCQ can produce reliable craving constructs, although more research is needed to evaluate the construct and concurrent validity of these three factors in both treatment and non-treatment seeking individuals who use opioids.

With respect to the original HCQ factors, the Lack of Self-Control factor aligned most closely with the original Lack of Self-Efficacy scale, whereas the Positive Expectancies factor aligned most closely with items from the original Desire subscale. Despite all four factors showing moderate to good test-retest reliability in individuals remaining abstinent across visits, only Desire and Self-Efficacy demonstrated acceptable internal consistency reliability, while Compulsivity and Relief showed unacceptable internal consistency. Moreover, three out of the four Compulsivity items and two out of the seven Relief items were not retained in the EFA. Taken together, Compulsivity and Relief do not appear to be as reliable as Desire and Self-Efficacy in this particular sample. Similar to newly extracted HCQ factors, at least three out of four original HCQ factors were positively related to the presence of comorbid AMP and negatively related to days since last heroin use. Also like the three newly extracted HCQ factors, none of the four original HCQ factors at baseline significantly distinguished between abstinent and returned to use groups at follow-up, suggesting that they may not be ideal in predicting future clinical outcomes.

While this study benefits from a deeply phenotyped sample of individuals with OUD and both cross-sectional and longitudinal analyses, there are several limitations that warrant discussion. First, this psychometric analysis focused on reliability, as an assessment cannot be valid unless it is reliable. Additional research is warranted to address the construct and concurrent validity of newly extracted HCQ factors and further address their clinical utility in predicting treatment outcomes for individuals with OUD. Second, this is a naturalistic study of individuals enrolled in treatment at baseline, as opposed to a clinical intervention study, thereby limiting conclusions that can be drawn regarding the clinical utility of HCQ factors. Moreover, due to (1) the treatment-seeking nature of our OUD sample and (2) the fact that over two-thirds of individuals in the study were prescribed buprenorphine, individual HCQ item scores were positively skewed towards lower craving levels. This restricted range may differ from non-treatment seeking samples, who may show greater variability in item responses and craving experiences. For instance, a recent systematic review of randomized controlled clinical trials suggests that buprenorphine is associated with lower craving than placebo but not methadone (Baxley et al., 2023). Although MOUD status was not a robust predictor of craving across factors, our results could be limited by imbalanced MOUD group sample sizes. Importantly, the treatment-engaged nature of our sample may have also contributed to differences in factor structure compared to prior studies. Discrepancies in factor solutions may reflect meaningful differences in the clinical state, treatment status, or stage of recovery of the sample, raising the possibility that craving measures may need to be adapted for specific populations or clinical contexts. Third, our OUD sample has significant clinical comorbidities, and we show that past-year AMP consistently related to higher HCQ factor scores. Therefore, these findings may not generalize to samples presenting with OUD as their sole diagnosis. However, research suggests that comorbidity between OUD and AMP is becoming more common (Santo et al., 2024; Zhu et al., 2021).

Fourth, there are limitations in study design and sample size regarding return to use. While the use of a broad definition of return to use, which included any substance or alcohol use, reflects the high rates of polysubstance use in individuals with OUD and enhances ecological validity, it may reduce the specificity of conclusions drawn about the relationship between opioid-specific craving and rsubstance use resumption. In addition, individuals who returned to use did not complete further visits due to human subjects considerations (such as the potential for active opioid withdrawal or intoxication), limiting knowledge of how HCQ craving constructs may fluctuate more dramatically immediately before and after resumption of use. Our follow-up group analysis was also limited by modest sample sizes for abstinent and return to use groups, limiting statistical power. Therefore, these findings should be interpreted with caution and larger longitudinal samples are warranted to test relationships between HCQ factors and clinical outcomes. Finally, the HCQ provides a snapshot of craving at a specific timepoint. Although prior research has demonstrated that a single timepoint craving assessment can meaningfully predict future substance use (Vafaie and Kober, 2022), craving is a dynamic construct that fluctuates over time. As such, relying on a single assessment may introduce some limitations in predicting substance use across longer follow-up intervals.

In conclusion, our findings suggest that researchers and clinicians could potentially save time and reduce participant burden by reducing the HCQ to 30 out of the 45 original items while still extracting factors that are internally consistent and stable during early recovery from OUD. However, future research should investigate whether the removal of 15 HCQ items impacts participant responses to the remaining 30 items. Moreover, replication of 30-item HCQ factor structure, reliability analyses, and clinical associations is warranted in additional samples varying in treatment-seeking status and heroin abstinence duration.

## Supplementary Material

Supplemental Material

Appendix A. Supporting information

Supplementary data associated with this article can be found in the online version at doi:10.1016/j.drugalcdep.2025.112753.

## Figures and Tables

**Table 1:** Sample demographic and clinical variables (*n* = 128).

Variable	Level	N	%
Recruitment Site	GRAND Addiction Recovery	109	85
	Women In Recovery	19	15
Sex Assigned at Birth	Female	46	36
	Male	82	64
Race	American Indian	12	9
	Asian	1	1
	Black	6	5
	More Than One Race	19	15
	Other	1	1
	White	88	69
Ethnicity	Hispanic	11	9
	Non-Hispanic	117	91
Education	Some High School	29	23
	High School Graduate or GED	58	45
	Some College	37	29
	Bachelor’s Degree or Higher	4	3
Lifetime Opioid Use Sessions	Less Than 500	2	2
	500–999	17	13
	1000 or More	109	85
Drug Injection	Any Drug	108	84
	Opioids	103	80
Current Opioid(s) of Choice	Heroin	41	32
	Fentanyl	40	31
	Heroin and Fentanyl	38	30
	Fentanyl and Oxycodone	4	3
	Oxycodone	3	2
	Heroin and Oxycodone	2	2
DSM-5 Diagnoses	Past-Year Opioid Use Disorder	128	100
	Past-Year Alcohol Use Disorder	24	19
	Past-Year Amphetamine Use Disorder	80	63
	Past-Year Cannabis Use Disorder	31	24
	Past-Year Cocaine Use Disorder	8	6
	Past-Year Sedative Use Disorder	21	16
	Past-Year Binge Eating Disorder	1	1
	Bipolar Disorder Unspecified	1	1
	Past-Year Generalized Anxiety Disorder	3	2
	Lifetime Major Depressive Disorder	31	24
	Past-Year Panic Disorder	9	7
	Past-Year Posttraumatic Stress Disorder	6	5
	Past-Year Social Anxiety Disorder	6	7
	Past-Year Obsessive Compulsive Disorder	1	1
	Antisocial Personality Disorder	20	16
Medication	Buprenorphine*	91	71
	Methadone	4	3
	Antibiotic	12	10
	Anticonvulsant	28	22
	Antidepressant	60	48
	Antihistamine	24	19
	Antihypertensive	27	21
	Antipsychotic	26	21
	Benzodiazepine	12	10
	Buspirone	14	11
	Naltrexone	6	5
Variable	Mean (SD)	Median	Range
Age (Years)	32.45 (6.39)	32.50	20–51
Heroin Abstinence (D)^#^	277.38 (450.89)	81.00	26–2952
Pre-Treatment Abstinence (D)^##^	27.66 (46.70)	8.00	0–236
Time in Treatment (D)**	36.86 (19.64)	31.00	14–141
Age First Opioid Use (Y)	17.97 (5.29)	16.00	8–36
Age Regular Opioid Use (Y)	19.73 (5.61)	19.00	8–38

Note.

* With or without naloxone.

** Data available for 121 participants.

# Data available for 125 participants.

## Data available for 127 participants. (D) = measured in days. (Y) = measured in years. GED = general educational development. DSM-5 = diagnostic and statistical manual for mental disorders, 5th edition.

**Table 2:** Regressions predicting extracted heroin craving questionnaire (HCQ) factors.

	Lack of Self-Control			Positive Expectancies			Urgency		

Overall Model	F(8, 112) = 4.92, *p* < .001*			*F*(8, 112) = 4.25, *p* < .001*			*F*(8, 112) = 3.51, *p* = .001*		
Adjusted R^2^	.21			.18			.14		
Outliers removed	4			4			4		
Residuals (Range)	[−1.76, 1.92]			[−1.86, 1.92]			[−1.37, 2.21]		
Individual Predictors	***β***	***t***	***p***	***β***	***t***	***p***	***β***	***t***	***p***
Age (Years)	− .01	− 0.13	89	.16	1.80	.07	.13	1.60	.11
Sex (1 = Female, 0 = Male)	.19	2.16	.03*	.06	0.65	.52	− .06	− 0.74	.46
Completed High School (1 = Yes, 0 = No)	− .04	− 0.47	.64	.11	1.26	.21	− .11	−1.43	.15
AMP (1 = Yes, 0 = No)	.31	3.76	< .001*	.26	3.20	.002*	.16	2.11	.04*
MOUD (1 = Yes, 0 = No)	− .12	−1.47	.14	− .07	− 0.87	.39	.09	1.11	.27
MDD (1 = Yes, 0 = No)	.09	1.01	.31	.17	1.96	.05	.03	0.39	.70
Days Since Last Heroin Use	− .23	− 2.71	.008*	− .25	− 2.90	.004*	− .14	−1.75	.08
ASI-3 Score	.22	2.48	.01*	.13	1.55	.12	.23	2.86	.005*

**Note**. Participants with missing data for days since last heroin use (*n* = 3) were not included in these regressions. AMP = amphetamine use disorder. MOUD = medication assisted treatment for opioid use disorder. MDD = major depressive disorder. ASI-3 = Anxiety Sensitivity Index 3. For all regression analyses, group ’0’ serves as the reference category.

**Table 3:** Regressions predicting original heroin craving questionnaire (HCQ) factors.

	Desire			Lack of Self-Efficacy			Relief			Compulsivity		

Overall Model	F(8, 109) = 4.02, *p* < .001*			*F*(8, 112) = 3.42, *p* = .001*			*F*(8, 111) = 3.96, *p* < .001*			*F*(8, 111) = 7.92, *p* < .001*		
Adjusted R^2^	.17			.14			.17			.32		
Outliers removed	7			4			5			5		
Residuals (Range)	[−1.85, 1.91]			[−1.81, 1.85]			[−1.59, 2.11]			[−1.78, 1.66]		
Individual Predictors	***β***	***t***	***p***	***β***	***t***	***p***	***β***	***t***	***p***	***β***	***t***	***p***
Age (Years)	.13	1.53	.13	.00	0.02	.98	.05	0.66	.51	− .18	− 2.40	.02*
Sex (1 = Female, 0 = Male)	.03	0.35	.73	.15	1.66	.10	.02	0.18	.86	.20	2.42	.02*
Completed High School (1 = Yes, 0 = No)	.07	0.81	.42	.03	0.41	.68	− .10	−1.31	.19	.21	2.77	.01*
AMP (1 = Yes, 0 = No)	.22	2.78	.006*	.27	3.32	.001*	.23	2.98	.004*	.09	1.19	.24
MOUD (1 = Yes, 0 = No)	.06	0.73	.47	− .03	− 0.34	.73	− .00	− 0.04	.97	− .21	− 2.76	.01*
MDD (1 = Yes, 0 = No)	.10	1.14	.26	.18	2.07	.04*	.23	2.85	.005*	.03	0.41	.68
Days Since Last Heroin Use	− .17	− 2.01	.046*	− .20	− 2.25	.03*	− .16	−1.99	.049*	− .46	− 5.89	< .001*
ASI–3 Score	.25	2.94	.004*	.14	1.59	.12	.16	1.94	.05	.11	1.43	.16

**Note**. Participants with missing data for days since last heroin use (*n* = 3) were not included in these regressions. AMP = amphetamine use disorder. MOUD = medication assisted treatment for opioid use disorder. MDD = major depressive disorder. ASI-3 = Anxiety Sensitivity Index 3. For all regression analyses, group ’0’ serves as the reference category.

**Table 4:** Intraclass correlation coefficients between visits for heroin craving questionnaire (HCQ) factors. Icc interpretation (Moderate = between.50-.75, Good = between.75-.90) was based on recommended cutoffs by koo and li (2016).

	Visit 1 to Visit 2 (*n* = 61)	Visit 1 to Visit 3 (*n* = 53)	Visit 1 to Visit 4 (*n* = 33)

Days Between Visits	Median = 37 Range = 24–105	Median = 73 Range = 56–189	Median = 111 Range = 81–238
Extracted HCQ Factors			
Lack of Self-Control	77 [.61,.86] Good	.59 [.29,.76] Moderate	.70 [.39,.85] Moderate
Positive Expectancies	.82 [.70,.89] Good	.72 [.52,.84] Moderate	.76 [.51,.88] Good
Urgency	.68 [.47,.81] Moderate	.54 [.20,.73] Moderate	.60 [.20,.80] Moderate
Original HCQ Factors			
Desire	.78 [.64,.87] Good	.69 [.47,.82] Moderate	.66 [.31,.83] Moderate
Lack of Self-Efficacy	.78 [.64,.87] Good	.57 [.26,.75] Moderate	.59 [.17,.80] Moderate
Relief	.68 [.46,.81] Moderate	.56 [.23,.74] Moderate	.59 [.17,.80] Moderate
Compulsivity	.78 [.64,.87] Good	.70 [.48,.83] Moderate	.55 [.08,.78] Moderate

Note. Numbers within brackets reflect 95 % confidence intervals.
