# Supplementary material for: Reliability and clinical utility of heroin craving questionnaire factors in treatment-enrolled individuals with opioid use disorder
Source: Drug Alcohol Depend. Author manuscript; Available in PMC 2025 Nov 29. (PMC12664305; doi:10.1016/j.drugalcdep.2025.112753)

**Reliability and Clinical Utility of Heroin Craving Questionnaire Factors**

**in Treatment-Enrolled Individuals with Opioid Use Disorder**

April C. May, Ph.D.^1^, Breanna A. McNaughton-Long, M.P.H. ^2^, Chrysantha B. Davis, M.S.^2^,

Abigail J. Pleiman, M.P.H.^2^, Carmen Buchfink, B.S.^2^, Rayus Kuplicki, Ph.D.^2^, Hung-Wen Yeh, Ph.D.^3^,

Martin P. Paulus, M.D.^2,4^ & Jennifer L. Stewart, Ph.D.^2,4^

**Supplemental Material**

**Table S1.** Heroin Craving Questionnaire (HCQ) items not included in the three extracted factors from the exploratory factor analysis.

| Item | Content | Original HCQ Factor Loading |
| --- | --- | --- |
| 3 | I would feel less sick if I used heroin. | Relief |
| 5 | Using heroin would not sharpen my concentration. (R) | None |
| 8 | I am going to use heroin as soon as possible. | None |
| 9 | My aches and stiffness wouldn’t go away if I used heroin. (R) | None |
| 11 | The desire to use heroin feels overpowering. | None |
| 16 | If I tried a little heroin I could not stop. | Compulsivity |
| 21 | I don’t want to use heroin now. (R) | Lack of Self-Efficacy |
| 25 | Starting now I could go without heroin. (R) | None |
| 29 | I wouldn’t be able to control how much heroin I used if I had some. | Compulsivity |
| 32 | Hot and cold flashes wouldn’t get better if I used heroin. (R) | Relief |
| 35 | I have no desire for heroin now. | Lack of Self-Efficacy |
| 36 | I wouldn’t think more clearly if I used heroin. (R) | None |
| 39 | I crave heroin right now. | Desire |
| 42 | I have an urge to use heroin. | Desire |
| 44 | I could easily control how much heroin I used right now. (R) | Compulsivity |

Note. (R) = reverse scored item.

**Table S2.** Welch’s *t* tests comparing Visit 1 HCQ craving scores as a function of medication for OUD, site, and sex. Participants taking medication for OUD (buprenorphine and/or methadone) reported lower Compulsivity scores (*M* = 17.31, *SD* = 5.77) than participants not on these medications (*M* = 19.91, *SD* = 5.78).

| New HCQ Factors | OUD Medication | Site | Sex |
| --- | --- | --- | --- |
| Lack of Self-Control | *t*[61] = 1.27, *p* = .21 | *t*[26] = -0.91, *p* = .37 | *t*[98] = -1.29, *p* = .20 |
| Positive Expectancies | *t*[59] = 0.52, *p* = .61 | *t*[29] = -0.49, *p* = .63 | *t*[97] = 0.11, *p* = .92 |
| Urgency | *t*[43] = 0.45, *p* = .66 | *t*[24] = 0.42, *p* = .68 | *t*[87] = 0.84, *p* = .40 |
| Original HCQ Factors | **OUD Medication** | **Site** | **Sex** |
| Desire | *t*[50] = -0.09, *p* = .93 | *t*[25] = 0.35, *p* = .73 | *t*[90] = 0.55, *p* = .59 |
| Lack of Self-Efficacy | *t*[56] = 0.72, *p* = .48 | *t*[28] = -1.04, *p* = .31 | *t*[100] = -1.05, *p* = .30 |
| Relief | *t*[54] = 0.99, *p* = .33 | *t*[27] = 0.70, *p* = .49 | *t*[95] = -0.09, *p* = .93 |
| Compulsivity | *t*[53] = 2.20, *p* = .03* | *t*[23] = 0.16, *p* = .88 | *t*[81] = -0.56, *p* = .58 |

**Note.** Welch’s *t* calculates degrees of freedom differently than the standard t-test because it accounts for unequal variances between groups.

**Figure S1.** Histograms of individual Heroin Craving (HCQ) item distributions (*N* = 128).


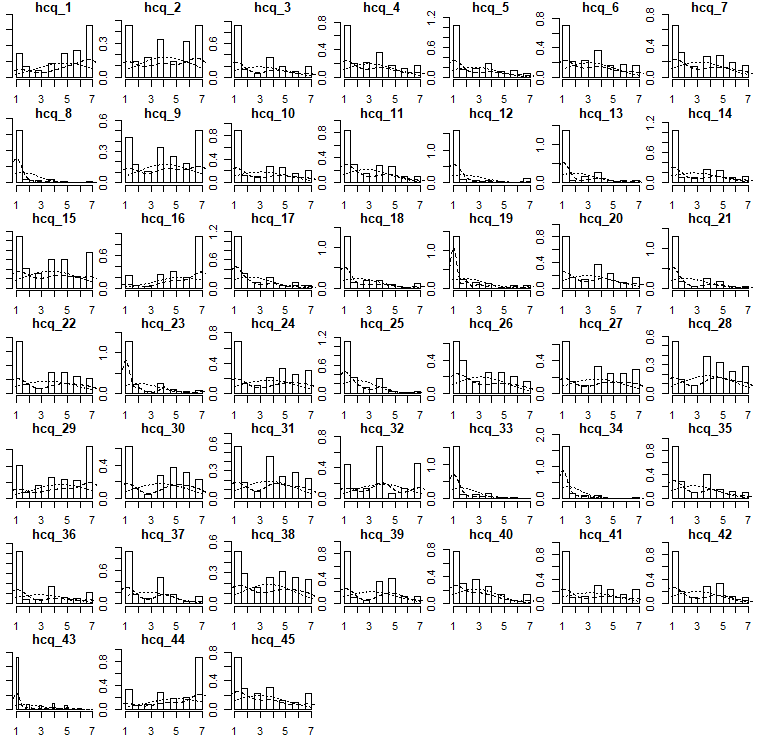


**Figure S2.** Exploratory factor analysis item loadings from baseline Heroin Craving Questionnaire (HCQ) data within participants from the GRAND site only (*N* = 109). (R) = reverse scored prior to factor analysis. Lines between items and factors indicate factor loadings, whereas darker lines between factors indicate correlations. The letter next to each item reflects its original HCQ factor loading: D = Desire, L = Lack of Self-Efficacy, R = Relief, C = Compulsivity, or N = None.


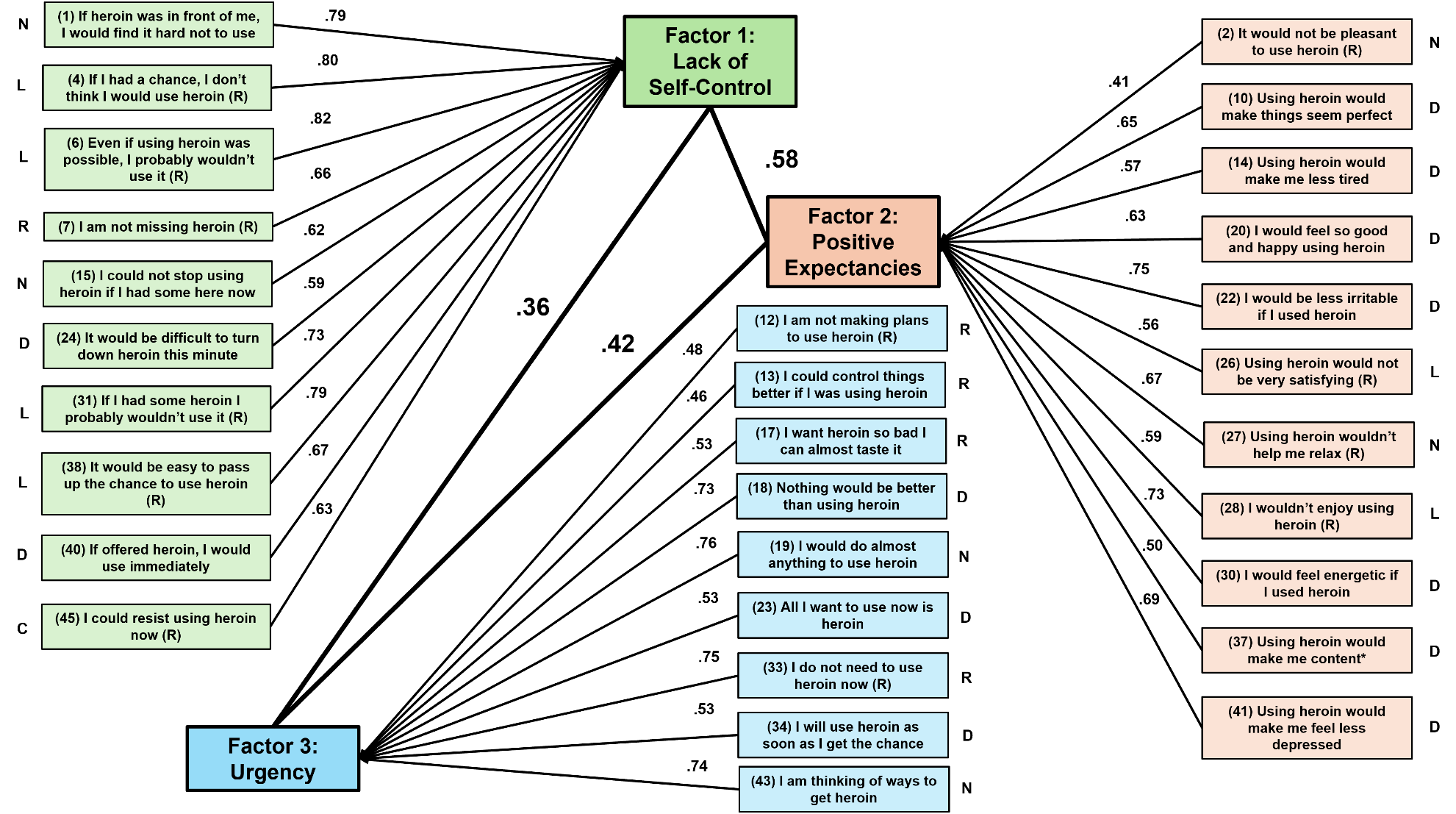


**Figure S3.** Extracted Heroin Craving Questionnaire (HCQ) factor scores at baseline as a function of follow-up group: Abstinent (*n* = 33) versus Relapsed (*n* = 44). Groups did not differ on any factor (all *p* > 18). Although statistics were computed on square root-transformed data for all factors, means and *+*1 standard errors depict raw data.


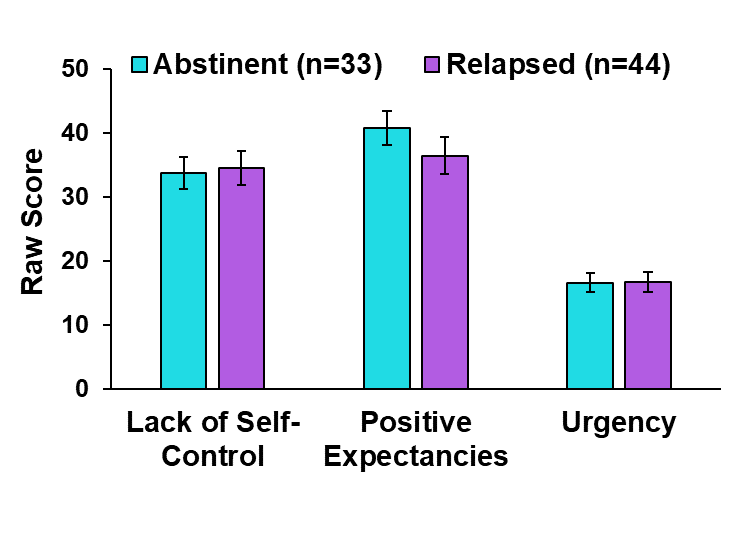


**Figure S4.** Original Heroin Craving Questionnaire (HCQ) factor scores at baseline as a function of follow-up group: Abstinent (*n* = 33) versus Relapsed (*n* = 44). Groups did not differ on any factor (all *p* > 51). Although statistics were computed on square root-transformed data for all factors, means and *+*1 standard errors depict raw data.


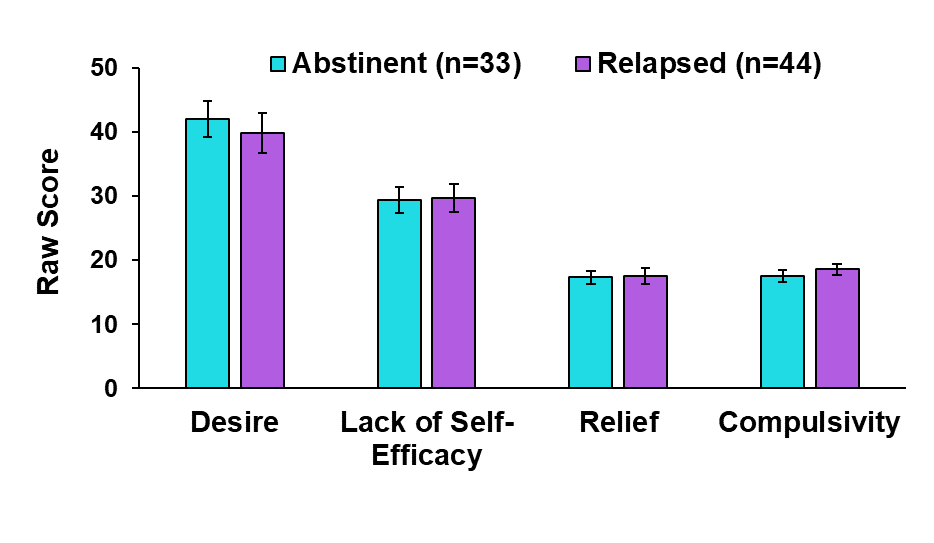


**Figure S5.** The original Heroin Craving Questionnaire (HCQ) factor scores as a function of visit (V1, V2, V3, V4) in participants who remained abstinent across visits (*n* = 33). Participants reported lower Desire scores at visits 2, 3, and 4 compared to visit 1 (*p* = .02, *p* = .007, and *p* = .004, respectively).

Error bars reflect +1 standard error. Asterisks indicate significant differences between visits.


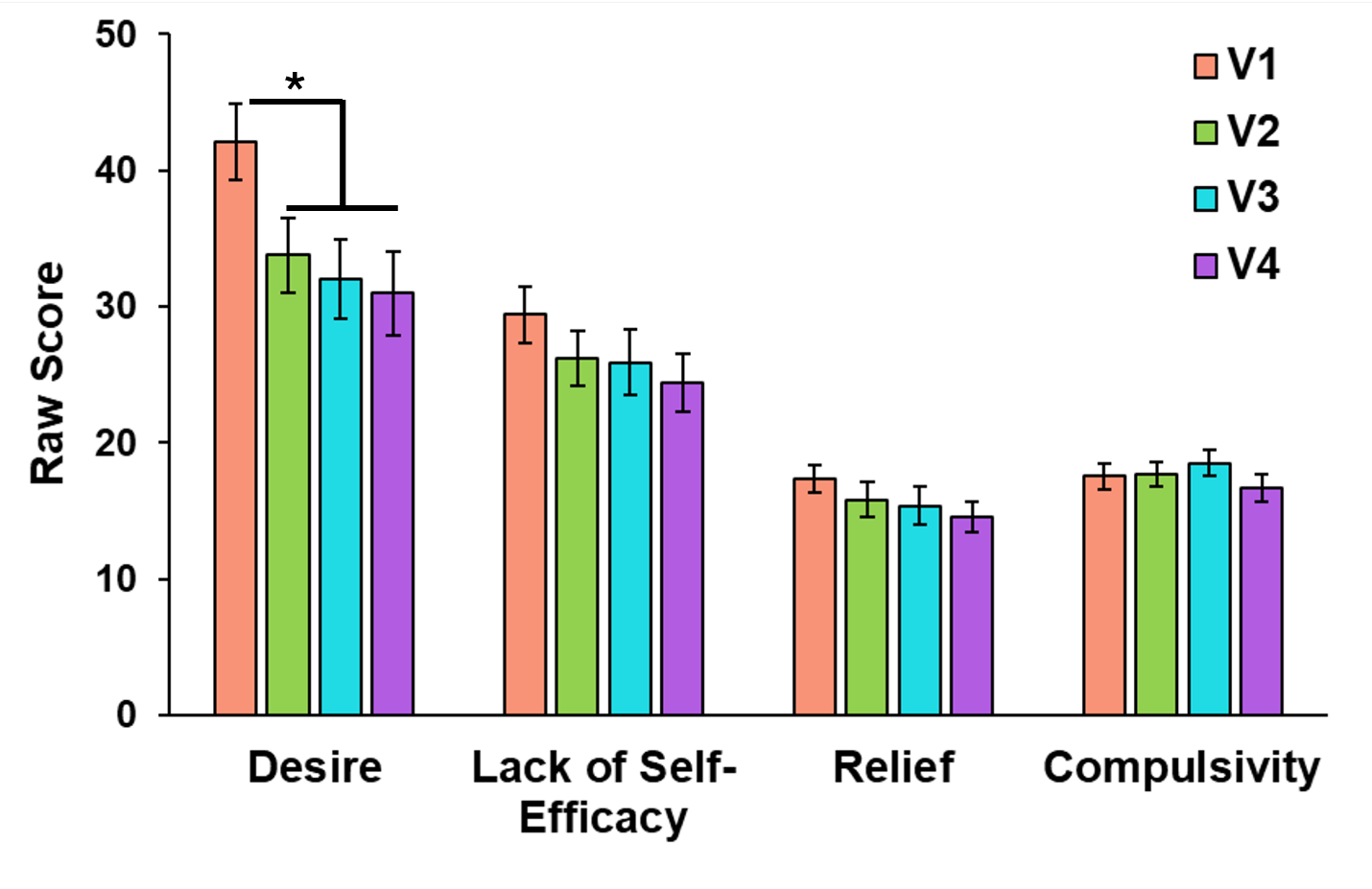

Supplement: Supplemental Material [file NIHMS2114592-supplement-Supplemental_Material.docx]
